# Verifying unfamiliar identities: Effects of processing name and face information in the same identity-matching task

**DOI:** 10.1186/s41235-022-00441-2

**Published:** 2022-10-12

**Authors:** Anita Trinh, James D. Dunn, David White

**Affiliations:** School of Psychology, UNSW Sydney, Kensington, NSW 2052 Australia

**Keywords:** Facial recognition, Visual matching, Face perception, Cognition, Matching, Context, Unfamiliar faces, Document bias, Perceptual interference

## Abstract

Matching the identity of unfamiliar faces is important in applied identity verification tasks, for example when verifying photo ID at border crossings, in secure access areas, or when issuing identity credentials. In these settings, other biographical details—such as name or date of birth on an identity document—are also often compared to existing records, but the impact of these concurrent checks on decisions has not been examined. Here, we asked participants to sequentially compare name, then face information between an ID card and digital records to detect errors. Across four experiments (combined *n* = 274), despite being told that mismatches between written name pairs and face image pairs were independent, participants were more likely to say that face images matched when names also matched. Across all experiments, we found that this bias was unaffected by the image quality, suggesting that the source of the bias is somewhat independent of perceptual processes. In a final experiment, we show that this decisional bias was found only for name checks, but not when participants were asked to check ID card expiration dates or unrelated object names. We conclude that the bias arises from processing identity information and propose that it operates at the level of unfamiliar person identity representations. Results are interpreted in the context of theoretical models of face processing, and we discuss applied implications.

**Supplementary Information:**

The online version contains supplementary material available at 10.1186/s41235-022-00441-2.

## Significance statement

 Face-matching tasks for unfamiliar faces are prevalent in many important applied settings, for example, passport screening and security checkpoints. Existing research has identified a tendency for novices and professional staff in these settings to make “match” biases when presented with unfamiliar face pairs in identity documents. This “match” bias can have detrimental impacts on border and national security, such as allowing fraudulent identity documents to be processed. Understanding the mechanisms and causes of these biases enables future research to develop a means of mitigating these biases. Here, we found that individuals were more likely to conclude that an unfamiliar face pair is a “match” after being shown matching name information, even when this information was irrelevant for the face-matching task. This bias appears to be specific to matching name information, suggesting that it is related to the automatic construction of identity representations. This result has implications for the design of workflow systems in applied settings where people verify the identity of unfamiliar people.

## Background

Matching the identity of unfamiliar facial images is an important component of real-world identity verification and identity management, and human performance on these tasks has implications for forensic investigations, criminal trials, and security settings. In spite of this, face-matching errors are quite common with standard participant groups making 20–30% errors on average despite optimal viewing conditions (e.g. Bruce et al., 1999; Burton et al., 2010). More problematically, these error rates are observed in tests of practitioners who perform face matching in their daily work, for example in passport control, police and security settings (White et al., 2014, 2020).

Compounding these high error rates, recent work has shown that biases can be induced by extraneous visual elements that are often present in real-world tasks. For instance, participants are more likely to make a “same face” decision when one of two facial images in a face-matching task is embedded in a passport frame (McCaffery & Burton, 2016). A similar “match” bias has also been observed in other forms of photo ID, such as driving licenses and student ID cards (Feng & Burton, 2019). This initial research suggests that contextual information can negatively impact the outcome of unfamiliar face-matching decisions, either through the reduction in overall face-matching accuracy or the generation of response biases.

The underlying causes of contextual information bias on face matching have not been explored systematically. However, early evidence appears to suggest that the presence of biographical information is an important factor. For example, while the validity of biographical information presented on an ID card does not impact face-matching accuracy (McCaffery & Burton, 2016), removing the biographical information from the ID card appears to remove the match bias (Feng & Burton, 2019).

Understanding why biographical information has been found to trigger the “match bias” is critical for applied settings. For example, when processing passport applications, staff often have to review biographical information like names, addresses and date of birth to determine whether they match existing records. Similar parallel processing of identity cues is common in other settings, for example police investigation, and so it is practically important to understand perceptual and cognitive causes of bias in face-matching decisions.

The question of how biographic information is interactively processed with perceptual information is also important theoretically. The Interactive Activation and Competition (IAC) model (Burton et al., 1990) provides a mechanistic account of how face and other personal information may be aggregated in person identity judgments. Although the IAC is intended to model the representation of familiar people, it can also be adapted to explain how unfamiliar identities are mentally represented. Central to this model is the idea of a “person identity node” (PIN) that aggregates input received from perceptual face information, identity-level details (such as a person’s name), and semantic information (such as a person’s nationality or occupation). Identification decisions occur at the level of PINs, with pooled activation from semantic, name, and perceptual inputs producing person recognition once a certain threshold of PIN activation is achieved. This means that the likelihood of recognising a familiar face is increased when a familiar name is mentioned, or when the face is presented with semantic information associated with the identity, for example when the US president appears beside an American flag.

Associative identity networks akin to the IAC model could also influence the processing of unfamiliar faces in applied settings. A recent review of neuroscientific evidence suggests that networks of brain areas responsible for encoding semantic and perceptual person information are both activated when we initially encounter faces (Kovacs, 2020; see also Shoham et al., 2021, Todorov et al., 2007). In addition, experiments by Menon et al. (2015) show that the formation of identity representations of unfamiliar faces are influenced by linking with identity labels (see also Dunn et al., 2021), and Schwartz and Yovel (2016) found improved facial recognition accuracy when unfamiliar faces are associated with name labels during learning. Together, this evidence suggests that the processing of identity-specific details in real-world identity verification tasks, such as name information, is likely to influence concurrent face-matching performance.

Here we report a series of experiments that were designed to test whether matching names biases subsequent face-matching decisions, and whether the mechanisms of such a bias align with an associative identity network account. Four experiments were designed to measure these biases, examine whether they operate at the level of person identity representations (Experiment 2) and identify whether they are also induced by tasks that require matching non-biographical information present on identity cards (e.g. card expiry date, Experiment 4).

We also examined whether the strength of contextual biases is modulated by the quality of perceptual information (Experiments 1–3). Perceptual ambiguity can be introduced to unfamiliar face images in multiple ways and often leads to decrease face-matching accuracy. Examples include pixelation (Bindemann et al., 2013), poor lighting (Johnston et al., 1992), and greater camera-to-subject distances (Noyes & Jenkins, 2017). However, to our knowledge, there is no existing research exploring how such decreases in facial image quality affect the processing of unfamiliar faces.

The processing of other perceptually ambiguous visual stimuli has consistently been found to correlate with increased activation in brain regions associated with top-down processing (Heekeren et al., 2008; Li & Yang, 2012; Maksimenko et al., 2020; also see Karimi-Rouzbahani et al., 2021). This increased activation may be interpreted as an increased reliance on contextual information to aid in disambiguating uncertain visual stimuli (e.g. Klink et al., 2012). A relationship between perceptual ambiguity and contextual reliance has been observed in object recognition (Oliva & Torralba, 2007), action recognition (Wurm & Schubotz, 2017), and across a range of different visual tasks (Dror et al., 2005; Qi et al., 2018). For instance, individuals are more likely to conform to the decisions of collaborators when performing perceptually difficult discrimination tasks (Qi et al., 2018). If unfamiliar faces are perceptually processed in a similar manner to other visual stimuli, we would expect an interaction between perceptual ambiguity and contextual biases. According to this prior work, the more perceptually ambiguous a facial image is to process, the greater the contextual bias should be. On the other hand, influential face processing models propose additive contributions of perceptual and semantic information in person identification decisions (Bruce & Young, 1986; Burton et al., 1990). If these contributions are additive as existing models suggest, then according to the logic of additive factors (see e.g. Sternberg, 1969, 2011), we would not expect to see an interaction between the amount of perceptual evidence and the effect of context.

Clearly, understanding how the magnitude of contextual biases interacts with image quality is important on a theoretical level in disentangling contrasting theories about how such an interaction occurs with unfamiliar faces. However, it is also important in an applied sense, because face identification decisions are often made based on low-quality CCTV images in criminal trials and investigations (Davis & Valentine, 2009; Edmond et al., 2010; Porter, 2009; Walker & Tough, 2015).

## Experiment 1

In Experiment 1, we examined two questions: firstly, whether matching name information biases subsequent face-matching decisions; and secondly, whether this bias is increased when there is greater perceptual uncertainty. We asked participants to complete sequences of name and face-matching decisions. For half of the total trials, the name pair type was consistent with the face trial type (e.g. if names matched then faces also matched) and in the other half it was inconsistent. We predicted that matching names would elicit a bias for participants to make more “match” responses to face pairs. Given that stronger effects of context have been observed when perceptual evidence is ambiguous (e.g. Dror et al., 2005; Qi et al., 2018; Wurm & Schubotz, 2017), we predicted that this bias would be stronger when face image quality was reduced for one of the two facial images.

### Method

#### Participants

Ninety-four undergraduate students from UNSW Sydney were recruited for Experiment 1. Sample size was based on Experiment 1 of the McCaffery and Burton (2016) study, the first known reported instance of the document bias, with additional participants to account for data exclusions. The data of one participant were removed because they did not complete the experiment, and two participants were omitted due to scoring below 85% accuracy on the name-matching task (task described in further detail below). A total of 91 participants (gender = 60 female, 30 male, 1 unspecified; M_age_ = 19.2 years, SD_age_ = 2.3 years) were included in the final analysis.

#### Stimuli

**Fig. 1:**
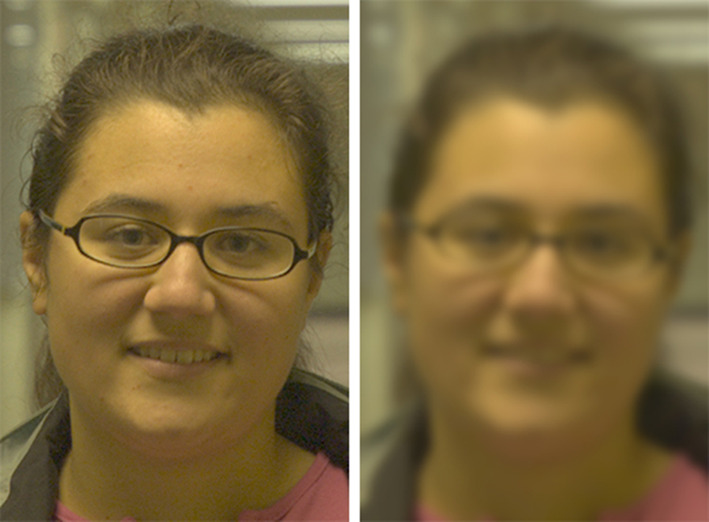
An example of a “high quality” facial image (left) and “low quality” facial image (right). The image on the right has been given a mosaic pixelation of 16 pixels in diameter using Adobe Photoshop

One hundred and sixty-eight image pairs were taken from the Expertise in Facial Comparison Test (EFCT), an unfamiliar face-matching test previously used to compare the accuracy of novices and professionals (White et al., 2015). Half of the image pairs show the same face (match pairs), and the remaining identity pairs show two different faces (non-match pairs). To manipulate image quality in our experiments, we downsampled one image in each pair in Adobe Photoshop using a mosaic pixelation with a 16-pixel diameter (i.e. 1/16 fewer pixels per cm). An example of low and high image quality photographs is shown in Fig. 1. Participants completed the experiment on a desktop computer with a monitor resolution of 1920 × 1080 pixels, and participants were seated approximately 60 cm away from the screen (approximately 50° visual angle). Image pairs were presented on-screen in colour at a size of 400 × 600 px (approximately 10.5 × 16 cm).

**Fig. 2:**
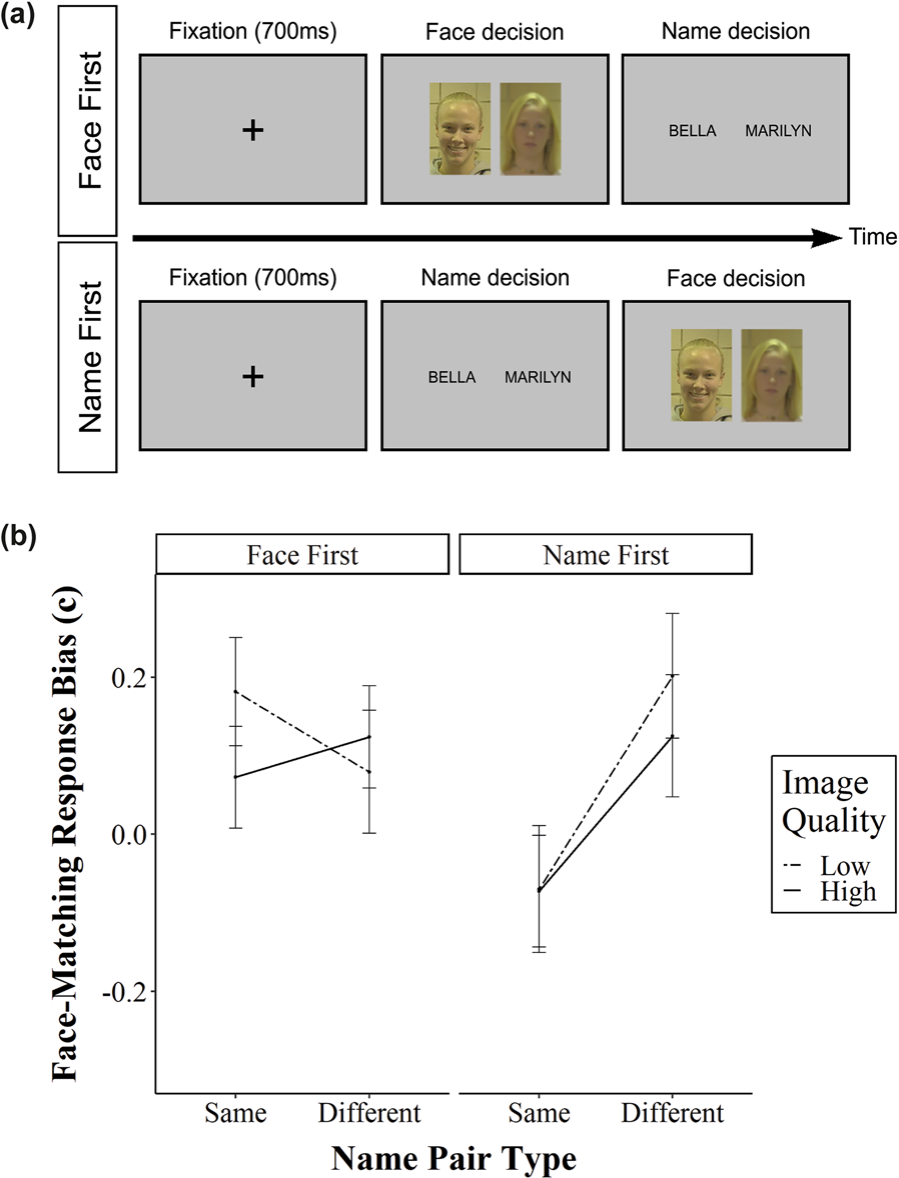
**a** An example of a trial sequence in Experiment 1. In the name-first condition (bottom), a fixation cross is presented, followed by a name-matching decision and a face-matching decision. In the face-first condition (top), the name decision and face decision in the trial sequence occur in the reverse order. Both trial sequences show a “different” name pair type. **b** Experiment 1 criterion scores across factors of image quality and name pair type, for both face-first and name-first conditions. All error bars represent standard error

Common first names were generated for each face to match the gender of the facial image; otherwise, the names were assigned at random. The names were presented in black capitalised Arial font. As shown in Fig. 2a, all names in the name task and facial images in the face task were displayed side-by-side and were equidistant from the centre of the screen (approximately 0.3° visual angle). All stimuli and written instructions were presented on a grey background.

#### Design and procedure

We used a 2 × (2 × 2) mixed factorial design, with Name Pair Type (same, different) and Image Quality (low, high) as the two within-subjects conditions. The Name Pair Type factor refers to whether name pairs in each trial are presented as matching or non-matching—“same” if the name pairs presented within a trial are matching, and “different” if the name pairs do not match. A factor of Task Order (name-first or face-first) was also included as a between-subjects condition, which modulated the order in which participants completed matching decisions for each identity (i.e. either matching name pairs first before face pairs, or vice versa). We would not expect face-matching decisions to be biased by name information if they were presented before the name-matching task, and so this task order provided a control condition. Participants were randomly allocated to one of the task order conditions—however, due to participant exclusions on the basis of name-matching performance accuracy, there was an unequal distribution of participants across the face-first (*n* = 45) and name-first conditions (*n* = 46).

The experiment was programmed using PsychoPy 3.0 (Peirce & MacAskill, 2018). Participants were instructed to assume the role of a surveillance officer checking the identities of employees who were entering and exiting a work building. Within this scenario, participants were required to check each presented identity against “database records” in a security system by indicating whether pairs of names and faces presented on-screen were of the same person or different people. Participants were instructed to make their decisions as accurately as possible.

Examples of experimental trials are shown in Fig. 2a. For each trial, participants were first shown a black fixation cross for 700 ms, followed by either name or face pairs (depending on task order allocation). For each name and face decision in a given trial, participants were instructed that they were to indicate “match” or “non-match” via key press. Two different pairs of keys were used to prevent accidental presses of the same key for separate decisions (“E” and “C” for the first matching decision; “I” and “M” for the second matching decision). The stimuli pair was displayed on-screen until the participants entered a valid keyboard input. Upon completion of the first matching decision, the stimuli pair for the second matching decision would immediately appear on-screen.

Each participant completed 168 trials. Participants received two short breaks after each third of the trials had been completed. There were an equal number of match and non-match face pairs in the experiment and match and non-match face pairs were equally likely to follow match and non-match name pairs and vice versa.

We originally planned for trials to be split equally across image quality conditions (84 high image quality, 84 low image quality). However, a coding error resulted in an unequal distribution across iterations of the experiment. The first 39 participants received 88 trials with low image quality and 80 trials with high image quality. The remaining 53 participants received 80 trials with low image quality and 88 trials with high image quality.

### Results

We analysed face-matching performance using signal detection measures of sensitivity and criterion (Stanislaw & Todorov, 1999). Two-way ANOVAs with factors of image quality (low, high) and name pair type (match, non-match) were conducted separately for the name-first and face-first conditions. Analysing results in this way enabled us to separate changes in perceptual discrimination (indexed by the sensitivity measure, d-prime) from changes in response biases (indexed by criterion). Because the main purpose of the name-matching task was to ensure participants processed name information, name-matching performance data were analysed only for the purposes of participant-level exclusions and an overall accuracy calculation. Name data are not analysed further within current and subsequent experiments.

Because we were interested in the biasing effect of name matching on face-matching decisions, our discussion here focuses primarily on face-matching criterion. Across all experiments, we found a consistent and expected main effect of image quality on sensitivity, in that a lower image quality led to significantly reduced sensitivity scores. As there were no other effects of note, we report all details on sensitivity scores in Additional file 1. On average, participants scored 77.7% accuracy on face-matching decisions (SD = 6.9%). The average performance across all participants in the name-matching decision was 98.0% (SD = 2.1%).

Criterion scores for the name-first and face-first conditions are shown in Fig. 2b. Name pair type had a large and significant effect on face-matching response biases in the name-first condition (F_1, 45_ = 33.35, *p* < 0.005, ηp^2^ = 0.43) which was not present in the face-first condition (F_1, 44_ = 0.38, *p* = 0.54, ηp^2^ = 0.01). The direction of this effect aligns with our predictions; participants in the name-first condition were more likely to make a “match” response for a face-matching decision when it was preceded by matching name pairs than when it was preceded by a pair of non-matching names. Image quality did not shift response biases in either condition (face-first: F_1, 44_ = 0.51, *p* = 0.48, ηp^2^ < 0.01; name-first: F_1, 45_ = 0.76, *p* = 0.39, ηp^2^ = 0.02), and the interaction between name pair type and image quality was non-significant for both conditions (face-first: F_1, 44_ = 3.34, *p* = 0.07, ηp^2^ = 0.07; name-first: F_1, 45_ = 1.01, *p* = 0.32, ηp^2^ = 0.02), showing that effects of name decision on the face-matching task were not modulated by image quality.

In the above analysis, we found clear evidence that when name-matching decisions preceded the face-matching task, there was a bias in participant responses. This pattern was observed only for the name-first condition. It came to our attention that, due to our experimental design, participants may have experienced the experiment as one continuous stream of unrelated matching decisions, as opposed to distinct trials with names assigned to facial identities. We hypothesised that if this were the case, *all* name decisions would bias subsequent face-matching decisions, regardless of which facial identity the name decision pertained to.

To test this possibility, we performed a post hoc analysis using data from the face-first condition where the face-matching decision had been preceded by a name-matching decision *in the previous trial*. We then conducted a 2 × 2 ANOVA for the face-first condition face-matching trials with factors previous name pair type (same, different) and image quality (high, low). We found a significant main effect of previous name pair type on response biases (F_1, 44_ = 5.83, *p* = 0.02, ηp^2^ = 0.12) whereby participants completing the face-first condition were more biased towards making a “match” face decision when the previous identity trial presented matching names. In other words, the name decision bias was not bound to singular trials. Results of this post hoc analysis are visualised in Additional file 1: Figure S5.

### Discussion

In our first experiment, we found that face-matching decisions were biased by prior name-matching decisions. In other words, participants were more likely to state that face pairs were a “match” when they were previously shown matching name pairs. Interestingly, this tendency did not interact with image quality, as has been observed in previous studies where context has been found to have a significant biasing effect on perceptual decisions.

Another unexpected result was that the response bias was not confined within individual experimental trials; the post hoc analysis revealed that name-matching decisions biased the decision of subsequent face-matching trials, regardless of the facial identity to which the names were assigned. In our second experiment, we aimed to test whether it was possible to restrict the locus of the bias observed in Experiment 1 to a single identity decision. To do this, in Experiment 2 we include a mock ID frame design that binds name and face information for each trial. We also aimed to minimise the possibility that participants were consciously altering their response behaviour because they believed that matching faces were more likely after matching names. Hence, we emphasised instructions that explicitly instructed participants that the name information should not inform face-matching decisions. Finally, we replaced key-press responses with mouse clicks as the decisional input.

## Experiment 2

### Method

#### Participants

Sixty-seven undergraduate participants from UNSW Sydney were recruited for the study. The participant count was based on a power analysis, using G*Power 3 (Faul et al., 2007), of detecting an effect size of ηp^2^ = 0.12. The effect size figure was obtained from a pilot version of this study (details of the pilot contained in Additional file 1).

After excluding one participant due to non-completion and one participant for performing under 85% accuracy in the name-matching task, sixty-five participants were included in the final analysis (gender = 47 female, 18 male; M_age_ = 19.5 years, SD_age_ = 2.9 years).

#### Stimuli

**Fig. 3:**
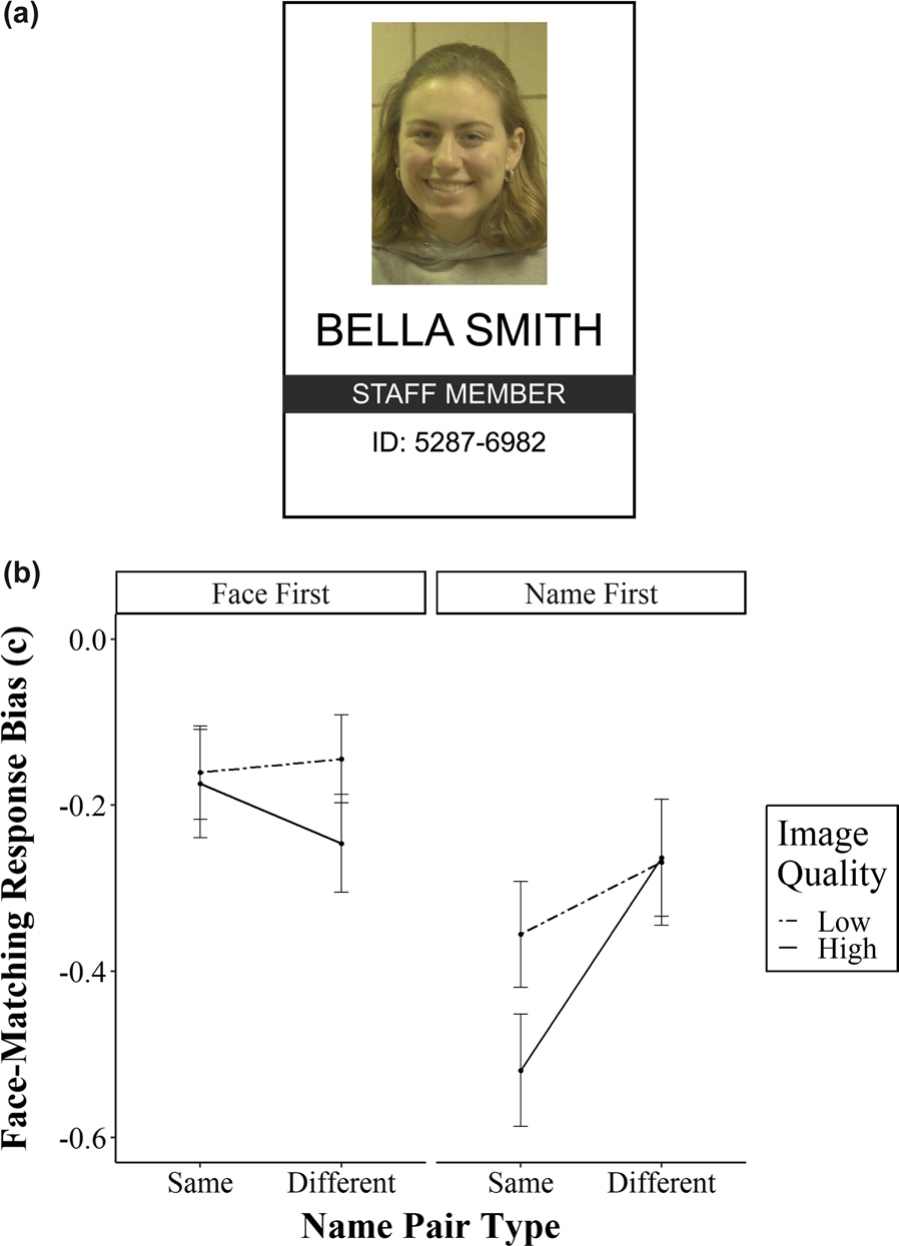
**a** An example of an ID card frame introduced in Experiment 2. ID card frames included a name and face, as well as a “STAFF MEMBER” title and a randomly generated ID number. **b** Experiment 2 criterion scores across factors of image quality and previous name pair type for both face-first and name-first conditions. All error bars represent standard error

Face stimuli were the same as the previous experiment, but names used in Experiment 2 contained both first and last names. To connect name and face-matching decisions more clearly to a singular identity representation, we embedded name and face information within a mock photo ID card containing a randomly generated ID number with the words “STAFF MEMBER” in black capitalised font. The ID cards and information from the “database” (either name or face, depending on the decision) appeared side-by-side on-screen so that participants could check database details against the ID information. An example of the ID frame is shown in Fig. 3a.

#### Design and procedure

The design of the experiment was the same as Experiment 1, with between-subject factor Task Order (name-first or face-first) and within-subjects factors Name Pair Type (same, different) and Image Quality (low, high). It was ensured that faces and names were allocated equally across conditions of name pair type, face trial type, and image quality. Due to randomisation and participant exclusions, there was a marginally unequal number of participants across the two between-subjects conditions (face-first: *n* = 35, name-first: *n* = 30).

For each trial, one of the two face images and names was embedded in the mock employee ID frame. Participants had to decide whether the face and name on the ID card matched the information in the “database” that appeared alongside. Participants also received additional instructions to those in Experiment 1 to avoid participants’ believing that name-matching decision was predictive of face-matching decision. They were told that a temporary worker had made random errors when entering employee names into the system and that they should not rely on employee names when making face-matching decisions.

For the name-matching task, a name was presented alongside the ID card and participants were required to respond “match” if the full names were identical. The first and last names shown on the ID card were randomly selected from a list of names that were culturally appropriate to the demographic of the face (e.g. “Wong” for a Chinese face). Where names did not match, the name pairs differed on either the first name or surname. For the face-matching task, participants decided whether the face on the ID card matched the identity of the database face presented alongside it. Responses were made via mouse click on buttons that said “same” or “different”. After each decision, participants were required to reset their cursor position by clicking a “continue” button that was equidistant from the same and different buttons.

We also conducted a pilot study that was very similar to Experiment 2. This pilot only used a name-first design, contained slight differences in the task instructions, and participants were given trial-by-trial feedback on their accuracy (“correct” or “incorrect”). The objective of this pilot was to examine whether the “match” bias for faces would persist in the name-first condition following the ID design change. The method and results of this study were very similar to Experiment 2 and so are presented in Additional file 1.

### Results

On average, participants performed at 79.7% accuracy for the face-matching decisions (SD = 8.6%). The average accuracy for participants in the name-matching decisions was 98.5% (SD = 1.3%).

Criterion scores are shown in Fig. 3b. Consistent with Experiment 1, there was a significant main effect of name pair type in the name-first condition (F_1, 29_ = 4.25, *p* = 0.048, ηp^2^ = 0.13), with participants more likely to make “match” responses for faces when they were preceded by matching names. There was no main effect of name pair type on criterion scores in the face-first condition (F_1, 34_ = 0.28, *p* = 0.60, ηp^2^ = 0.01), nor was there a main effect of image quality on face-matching response biases (face-first: F_1, 34_ = 1.00, *p* = 0.32, ηp^2^ = 0.03; name-first: F_1, 29_ = 1.53, *p* = 0.23, ηp^2^ = 0.05). There was no interaction between name pair type and image quality in either between-subjects condition (face-first: F_1, 34_ = 0.85, *p* = 0.36, ηp^2^ = 0.02; name-first: F_1, 29_ = 3.21, *p* = 0.08, ηp^2^ = 0.10).

We also repeated the post hoc analysis of the face-first group data from Experiment 1 to determine whether face-matching biases were also generated by name-matching decisions from subsequent trials. In contrast to the post hoc analysis in Experiment 1, we found no effect of previous name pair type on response bias for face-matching decisions (F_1, 34_ = 0.47, *p* = 0.50, ηp^2^ = 0.01). This finding leads us to two conclusions. Firstly, the face-matching biases observed in Experiment 2 are only being generated by name pairs that precede the face decision and pertain to the same identity. Secondly, the inclusion of an ID frame is likely the reason why name-matching decisions do not bias subsequent face-matching decisions in the face-first condition (which is what was observed in Experiment 1). A figure of the post hoc analysis is shown in Additional file 1: Figure S6.

### Discussion

In Experiment 2, we incorporated name and face information into an ID frame and explicitly instructed participants not to rely on name information when making face-matching decisions. Even with these changes, we replicated the results of Experiment 1, as participants continued to show a significant bias towards a “match” response for face pairs that were preceded by same name pairs. Unlike Experiment 1, however, we found that face-matching biases were generated only by preceding name pairs pertaining to the *same* identity. Due to changes to instructions, it is unlikely that the bias is caused by participants consciously relying on name-matching information to predict face-matching decisions. Rather, our results suggest that the bias is a product of a more implicit cognitive mechanism, resulting from the binding of identity-specific details and facial information. We investigate the cognitive mechanisms of the decisional bias further in Experiment 4; Experiment 3 will continue our secondary investigation into whether image quality can influence the strength of face-matching response biases.

Consistent with Experiment 1, the results of Experiment 2 again found no interaction between image quality and name pair type on face-matching response biases. This appears to suggest that the mechanisms of the bias are independent of perceptual processing. However, we hesitate to conclude that this is the case without testing two alternative explanations. One possibility is that no interaction is present because no causal or predictive link was established between the name information and face-matching outcome. We previously mentioned that one function of incorporating context into ambiguous perceptual decision-making is to disambiguate or clarify one’s interpretation of the visual stimuli (see Maksimenko et al., 2020). Hence, if the contextual information is perceived as non-predictive, participants may not direct their attention to name information and utilise it when the task becomes perceptually more difficult.

A second possible explanation is that the presence of contextual information *during* a perceptually ambiguous task may be crucial to contextual reliance, as seen in other experimental paradigms with an interactive effect (e.g. Dror et al., 2005; Wurm & Schubotz, 2017). Ambiguity during perceptual decision-making may lead to an increased cognitive load, meaning that contextual information may not be relied upon in the decision-making process unless it is readily accessible. These two explanations are further tested in Experiment 3.

## Experiment 3

Experiment 3 modified the design of Experiment 2 to bring it closer to studies that have found an interaction between perceptual uncertainty and contextual biases. Firstly, we changed participant instructions to inform participants that the name-matching decision was somewhat predictive of whether the faces also matched. Secondly, we strengthened the context manipulation by retaining the name information under both face images when participants made face-matching decisions. We hypothesise that, following these design changes, we would observe an interaction between image quality and name pair type whereby the contextual bias observed for “same” name pair types would be more pronounced for the low compared to high-quality facial images.

### Method

#### Participants

Forty-three undergraduate students from UNSW Sydney participated in the study. All participants were recruited online and completed the study online on Pavlovia (Peirce & MacAskill, 2018). As there was no between-subjects condition in this experiment, the sample size was based on recruiting half the number of participants collected in Experiment 2, plus an additional ten participants to account for any data-based exclusions. We confirmed from a G*Power 3 analysis (Faul et al., 2007) that this resulting sample size allowed for sufficient power in detecting an effect size of at least ηp^2^ = 0.15 (effect size based on name pair type effect in previous experiments).

As the study was run online, we applied stricter exclusion criteria to the data and required that participants score equal to or above 95% on average name-matching and 50% on face-matching performance to be included in the analysis. One participant was removed because of having a low average name-matching score. Forty-two participants were included in the final analysis (gender = 32 female, 10 male; M_age_ = 19.7 years, SD_age_ = 5.0 years).

#### Stimuli

**Fig. 4:**
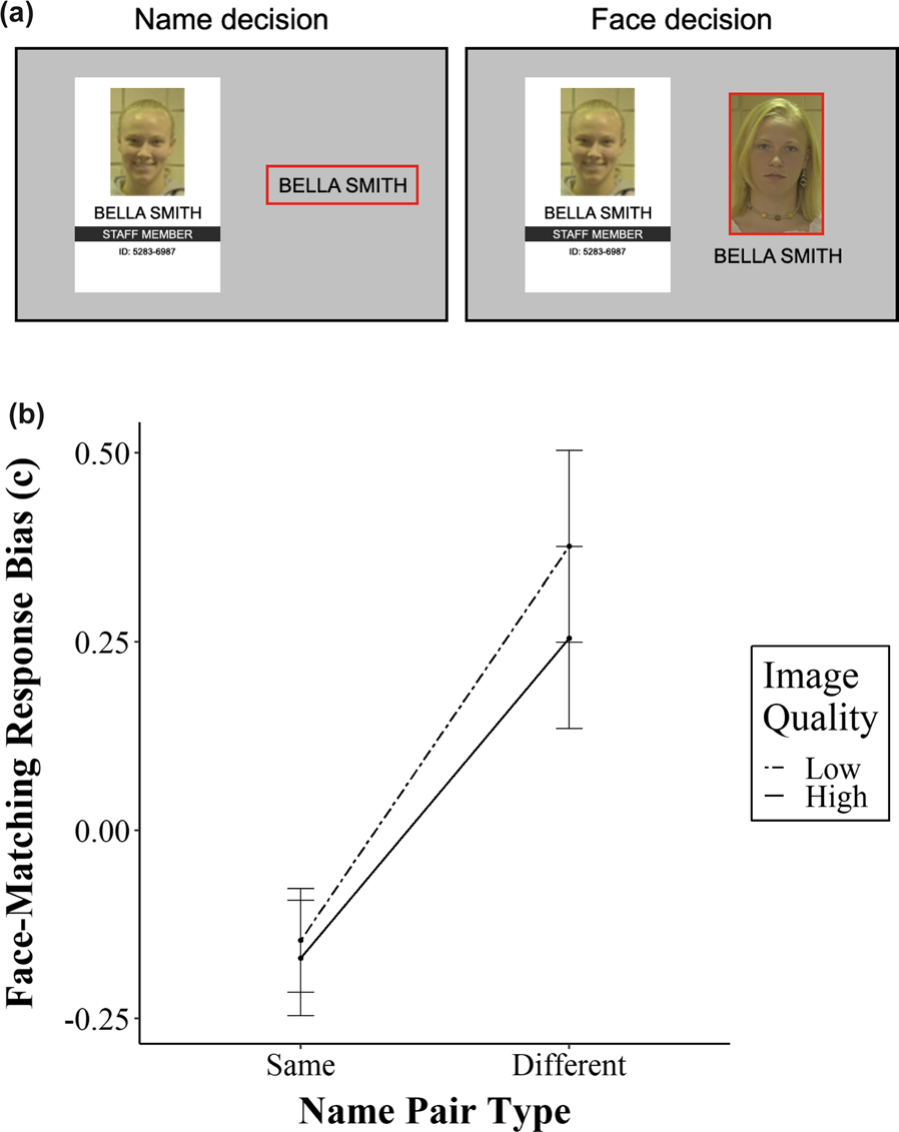
**a** A visual example of the red outline that participants viewed in Experiment 3 for name and face decisions within each trial. The outline was added to the “database” information to remind participants of which matching decision they were required to complete at each stage of the trial. **b** Experiment 3 criterion scores across levels of name pair type and image quality. All error bars represent standard error

The same facial and name stimuli used in Experiment 2 were used. The stimuli design was identical to that of Experiment 2’s “name-first” condition, except that participants were shown *both* the name and face information from the database during the face-matching task. A red box would outline the database information on the right side of the screen that participants were required to match in each individual response. A visual example of this red outline is shown in Fig. 4a.

#### Design and procedure

Experiment 3 had a 2 × 2 within-subjects design, with Name Pair Type (same, different) and Image Quality (high, low) as the factors. There were equal numbers of trials across all conditions.

The procedure is identical to that of Experiment 2’s “name-first” condition but with a modification to participant instructions. Participants were told that there was a greater likelihood of facial images matching when the names matched, and facial images being a non-match when the names were not matching.

### Results

On average, participants performed at 76.5% accuracy for the face-matching task (SD = 9.0%), and 98.5% accuracy for the name-matching task (SD = 0.1%).

Criterion scores are shown in Fig. 4b. We replicated the name decision response bias observed in our previous experiments, as shown by the significant main effect of name pair type on face-matching response bias (F_1, 41_ = 15.97, *p* < 0.001, ηp^2^ = 0.28). Again, participants were more likely to respond “match” to a face-matching task when previously presented with a same name pair type. Notably, our manipulations to participant instructions have made this effect larger than before (ηp^2^ = 0.13 in Experiment 2 compared to ηp^2^ = 0.28 in Experiment 3). However, as with the previous experiments, there was no main effect of image quality (F_1, 41_ = 2.50, *p* = 0.12, ηp^2^ = 0.06) and, most critically, no interaction between image quality and name pair type (F_1, 41_ = 1.04, *p* = 0.31, ηp^2^ = 0.02).

### Discussion

Consistent with the findings of the previous two experiments, participants were significantly more likely to respond “match” to a face-matching decision when it was preceded by a name-matching decision with matching names. However, despite the experimental changes implemented to maximise the likelihood of an interaction between image quality and name pair type, we still did not observe a significant interaction. While this is a consistent result across Experiments 1 to 3, it is contrary to findings with other perceptual stimuli (Dror et al., 2005; Qi et al., 2018). Interestingly, however, the result appears to be consistent with models that propose additive—rather than interactive—contributions of perceptual and semantic information in person identity decisions (Bruce & Young, 1986; Burton et al., 1990). The implications of this finding will be discussed further in our General Discussion.

In our next experiment, we refocus on the main effect of face-matching response bias induced by name information. Based on our observation in Experiment 2, we suspect that the biasing effect of name-matching decisions on face decisions is caused by an implicit cognitive mechanism, namely the development of person identity representations.

To empirically test this, we next examine whether making comparisons of *any* information—i.e. not person information *per se*—also induces the observed response bias. We test this in the next experiment by comparing biases induced by matching names to the biased induced by matching non-person information.

## Experiment 4

Previous experiments have consistently shown that name-matching decisions induced a face-matching response bias. However, it is not clear whether this bias effect operates at the level of person identity, or whether it reflects a more domain-general criterion setting change caused by matching *any* form of written information. In Experiment 4, we therefore test whether this same bias can be induced by making other types of matching decision that do not involve person information, for example from matching ID card expiry dates and names of common objects. Because image quality did not modulate bias in our previous experiments, we did not include this manipulation in our final experiment.

### Method

#### Participants

Eighty participants were recruited from Amazon Mechanical Turk and were financially reimbursed for their participation. The study was advertised as a psychological experiment involving matching tasks with faces and written information.

Based on a G*Power analysis (Faul et al., 2007), a sample size of 40 participants would have provided at least 80% power for detecting an effect size of ηp^2^ = 0.15 (effect size figure based on previously observed name pair type effect). However, we collected a larger sample size here to have sufficient power for a mixed-model ANOVA which tested a hypothesis beyond the scope of this paper (detailed in Additional file 1).

Seven participants were excluded from the analysis on the basis that they scored lower than 95% in context-matching accuracy (further details of context-matching in Design and Procedure) or below 50% in face-matching accuracy. Seventy-three participants were included in the final analysis (gender = 45 male, 28 female; M_age_ = 41.0 years, SD_age_ = 11.9 years).

#### Stimuli

The same facial images and names as those in Experiment 2 were used for the face-matching and name-matching tasks in this experiment, respectively. However, we also modified Experiment 2 by adding two different types of written information—ID card expiry dates and names of common objects (henceforth referred to as the “date” and “object” information types). Expiry dates assessed whether matching non-personal information commonly contained on ID cards can induce biases. Names of common objects were introduced to assess whether matching general written information can create face-matching response biases.

**Fig. 5:**
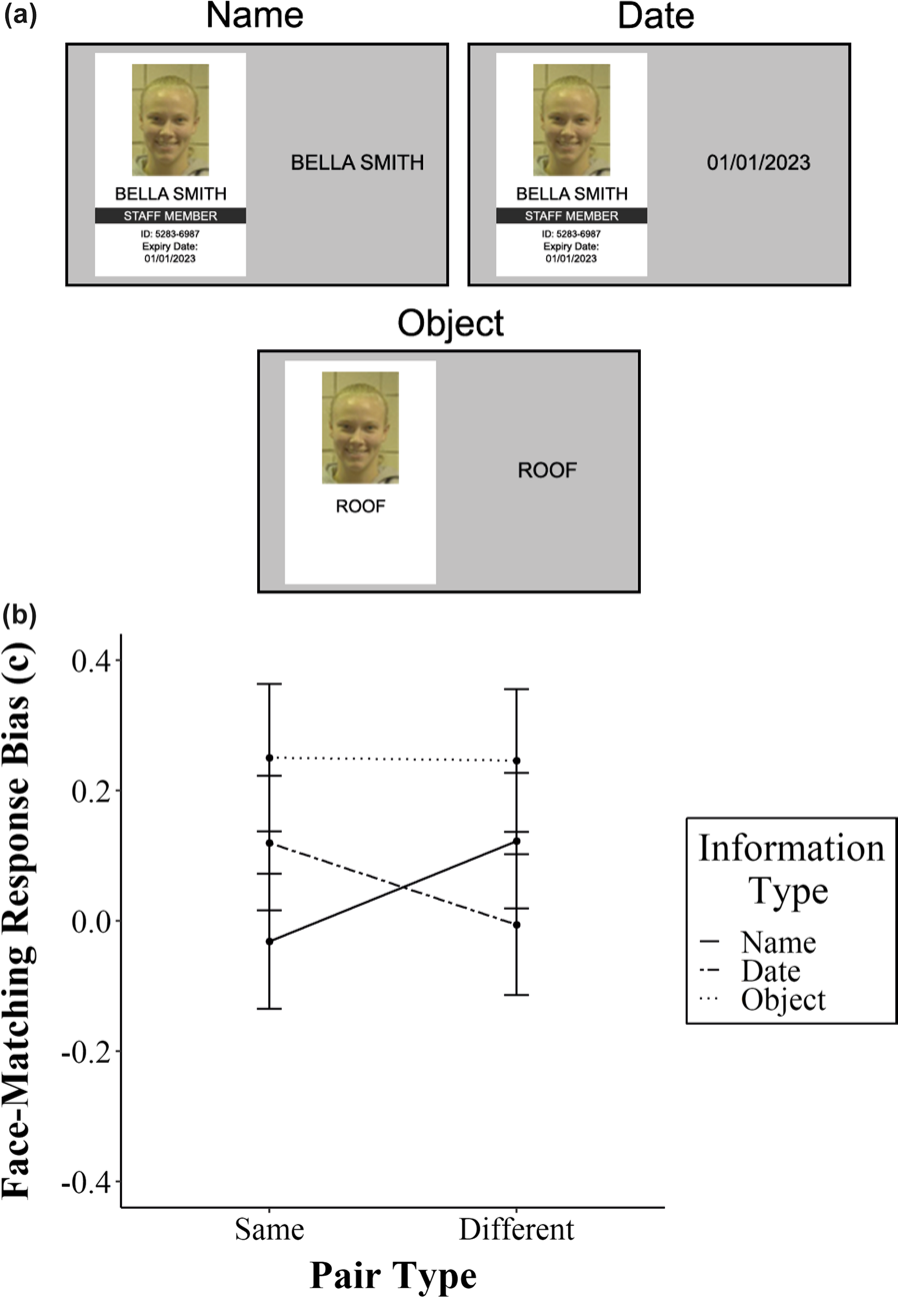
**a** Trial examples of the name, date, and object context pair types in Experiment 4. In the name and date pair type conditions, participants were shown an ID card similar to that in Fig. 3a, but for the addition of expiry date information. In the object-matching condition, participants were shown a white frame with an object label under the facial image. **b** Experiment 4 criterion scores as a function of context pair type and context condition. All error bars represent standard error

The ID card shown on-screen is identical for the name and date information types, as illustrated in Fig. 5a. The appearance of the ID card in these two conditions is highly similar to that shown in Fig. 3a, except for the inclusion of the words “Expiry Date” and a date in the format “DD/MM/YYYY” positioned below the ID number. In the object information condition, an object label is inserted where the name information would originally be (i.e. below the facial image) and no other written information is present. The object label and facial image are contained in a white frame identical in size and shape to that shown in Fig. 3a.

Expiry dates displayed on the ID card were randomly generated to be later than the dates on which the experiment ran. Non-match dates displayed on the right side of the screen, on the other hand, always preceded the dates presented in ID frames. Object labels were randomly selected from a list of 252 common concrete nouns obtained from the Internet. The list of concrete nouns was curated to avoid nouns that could have high emotional valance or have an implicit association bias. Some examples include “OCEAN”, “COMPUTER”, and “SHAMPOO” (for a comprehensive list of chosen objects, see Additional file 1).

#### Design and procedure

All participants in this experiment performed name, date or object-matching decisions prior to making face-matching decisions, and the three different types of decisions were made in separate blocks of 56 trials each (block order was counterbalanced across participants). Visual examples of these different decision types are shown in Fig. 5a. These changes resulted in a 2 × 3 factorial design, with within-subjects factors of Pair Type (same, different) and Information Type (name, date, object). The order in which participants completed information type matching blocks was systematically randomised such that an even number of participants completed the name, date, or object conditions first. This design choice was made based on results from an Experiment 4 pilot study, which had fully randomised the order in which participants completed the information type matching blocks. Further details about the Experiment 4 pilot study are included in Additional file 1.

Different instructions were shown prior to each block based on the context condition. Instructions preceding the name and date condition block were the same as that provided in Experiment 2 using the security surveillance scenario. For the object condition, participants were not given a security surveillance scenario, but were simply informed that they needed to check whether the object name and face on the left of the screen matched the object name and face shown on the right. Prior to all context conditions, participants were informed that they should not rely on written information when matching faces. Participants completed three practice trials with feedback prior to each trial block to become accustomed to matching each context type.

### Results

On average, participants performed at 73.1% accuracy for the face-matching task (SD = 24.7%), 98.2% accuracy for the name-matching task (SD = 4.3%), 99.4% accuracy for the date-matching task (SD = 2.3%), and 99.6% accuracy for the object-matching task (SD = 2.0%).

Criterion scores for Experiment 4 are shown in Fig. 5b. The overall main effect of pair type on face-matching criterion scores was non-significant (F_1, 70_ = 0.05, *p* = 0.83, ηp^2^ < 0.001). However, there was a significant two-way interaction between pair type and information type (F_2, 140_ = 4.86, *p* = 0.009, ηp^2^ = 0.06). Simple main effects show that while there was a matching bias after same name pairs in the predicted direction (F_1, 72_ = 5.16, *p* = 0.02, ηp^2^ = 0.07), the opposite pattern emerged after date matching (F_1, 72_ = 4.41, *p* = 0.039, ηp^2^ = 0.06). In other words, a “match” bias occurred for face-matching decisions after being shown *non-matching* date pairs. Finally, there was no significant shift in response bias found after object matching (F_1, 72_ = 0.004, *p* = 0.94, ηp^2^ < 0.001). This suggests that the matching bias replicated in this experiment only occurs after matching names but not after matching any other information.

### Discussion

Results of Experiment 4 replicated previous experiments but also indicate that the bias is linked to the processing of identity information. Matching names prior to matching faces led to a face-matching response bias consistent with the name pair type, but this shift in bias was not found when participants matched expiry dates or names of objects prior to face-matching decisions. This suggests that the bias in face-matching decisions we have observed in all our experiments arises from mechanisms that are primarily involved in the processing of person information.

## General discussion

Across four experiments, we examined the impact of matching name information on unfamiliar face-matching decisions using an experimental design similar to identity verification tasks in applied settings. We found consistent evidence that participants were significantly more likely to make “match” decisions for face pairs when face-matching decision were preceded by matching names. This bias remained even when participants were instructed *not* to rely on name information (Experiment 2) and was not found for matching decisions based on non-person information (Experiment 4).

Our work extends recent studies reporting a response bias in face matching from processing contextual information (Feng & Burton, 2019; Fysh & Bindemann, 2018; Howard et al., 2020; McCaffery & Burton, 2016; Robertson & Burton, 2021). We also provide evidence that this face-matching bias operates on an implicit level, because it was not eliminated by explicit instruction that the name information was uninformative to the face-matching decision.

Interestingly, we found that this bias was induced by name-matching decisions but not when processing information unrelated to person identities. This may suggest that the origin of the bias is the representations of person identities. More specifically, as individuals process unfamiliar faces in combination with identity-specific details, an unfamiliar face representation may be developed which incorporates these two sources of information (similar to that of a PIN from the interactive activation model; Burton et al., 1990) and influences subsequent decision-making pertaining to the face. This possibility is feasible, given the strong evidence that identity-based, top-down information is readily integrated into perceptual decisions about unfamiliar face identities (Dunn et al., 2021; Menon et al., 2015; see also Andrew et al., 2015), and the established finding that concurrent presentation of incongruent name or semantic information interferes with the processing of face identity (Bindemann et al., 2005; Jenkins et al., 2003; Young et al., 1986). We propose that the bias we observe here might stem from functional mechanisms that integrate semantic and perceptual information with unfamiliar faces to support the everyday learning and recognition of new facial identities (e.g. Ambrus et al., 2021; Bonner et al., 2003; Kaufmann et al., 2008; Schwartz & Yovel, 2016, 2019; Shoham et al., 2021).

Our experiments show that processing name information on ID cards caused biases in unfamiliar face identity processing, but processing document-based information such as expiry date did not. This suggests that identity-level representations may be contributing to the “document bias” of matching unfamiliar faces (Feng & Burton, 2019, 2021; McCaffery & Burton, 2016). However, it does not identify whether matching *other* forms of biographical information, such as an individual’s occupation, gender, or nationality, would induce the same face-matching bias examined across our experiments. According to the structure of the interactive activation and competition model (Burton et al., 1990), details that more uniquely identify an unfamiliar face representation may be more successful in forming an unfamiliar face representation (e.g. names as opposed to biographical details), and thus more likely to generate biases when processed prior to face matching. This is because name information is described as forming a part of a person identity node (PIN) upon which a person representation is based, while semantic details such as nationality form semantic information units (SIUs) are connected to the PIN in a one-to-many fashion (e.g. multiple person identities can share the same birthday or nationality). Future experiments will be required to test whether the bias we report is also caused by other sources of person information, or whether names are especially biasing—for example, in the light of strong name–face interference effects in face processing tasks (e.g. Bindemann et al., 2005) and a general tendency for people to form strong face–name associations (Ramon et al., 2016; Schwartz & Yovel, 2016).

Critically, our results demonstrate that the integration of perceptual information with sources of person information that are not informative for the task of identification can be detrimental to accuracy (Dunn et al., 2021; Menon et al., 2015). The biases in face-matching decisions observed here and in prior work (Howard et al., 2020) highlight a need for practical solutions to minimise face-matching biases in real-world tasks. The fact that explicit instructions to ignore name-matching decisions were ineffective in Experiment 2 suggests that these biases are implicit and may go unnoticed in applied settings. In future work, one approach to mitigating these biases would be to follow solutions that have been proposed in forensic pattern-matching domains. For example, in fingerprint matching (Dror et al., 2005, 2006; Smalarz et al., 2016), researchers have proposed a solution known as linear sequential unmasking (LSU; Dror et al., 2015). LSU is a process that involves providing only task-relevant information to the forensic scientist (e.g. the fingerprint pair), then providing other forms of information only when necessary to accurately make the matching decision.

Across our experiments, we consistently found that the biasing effect of name-matching decisions was not affected by the quality of images being compared. Even when name-matching decisions were presented on-screen alongside the face-matching decision, this remained the case. Our findings align with a prominent model of person identity representation (IAC; Burton et al., 1990) where the pooling of different sources of identity information is based on the additive accumulation in a central “hub”. This is opposed to mechanisms where there is a modulating effect of semantic context on perceptual representations themselves, as has been proposed for example by predictive coding models (see e.g. Trapp et al., 2018).

On the other hand, the absence of an interaction is contradictory to the results of researchers in other visual fields who observed an interaction between perceptual uncertainty and decision-making (Dror et al., 2005; Qi et al., 2018; Wurm & Schubotz, 2017). We do note, however, that the *form* of context utilised in this experimental paradigm differed in a qualitative nature from that of prior studies. While operationalisations of “context” in prior literature on ambiguous perceptual decision-making varied widely (see e.g. Klink et al., 2012), a majority of successful interactive effects we observed were based on operationalising and conveying “context” as visual information (e.g. scene information; Wurm & Schubotz, 2017). Written information, as utilised in our experimental paradigm, may not be coded with the same level of automaticity as visual contexts.

We tentatively hypothesise that, for written information in forensic contexts to interact with perceptual uncertainty, it may need to be as directly relevant to the perceptual decision-making as possible. A clear example of such written information, in face-matching paradigms, is AI labels which express the likelihood or opinion of whether an unfamiliar face pair is a match or not (e.g. as seen in Fysh & Bindemann, 2018). This possibility would be a promising avenue for future research, with direct practical implications for forensic contexts whereby expert reports and opinions of visual information are often paired alongside perceptually ambiguous stimuli.

## Conclusions

In summary, we have shown persistent biasing effects on face-matching decisions resulting from making concurrent checks of other identity information. These were robust to a variety of experimental design implementations and changes to task instructions. Should these biases transfer to real-world settings, they would have potentially profound consequences, for example, leading to the issuance of fraudulent identity documents or admitting imposters to secure access areas. Future research should therefore aim to better understand the causes of these biases and techniques to mitigate them.

## Supplementary Information

**Additional file 1.** Supplementary Materials.

## Data Availability

The data sets used and analysed during the current study are available in the Open Science Framework repository at the following link: https://osf.io/jhw25/.

## References

[CR1] Ambrus GG, Eick CM, Kaiser D, Kovacs G (2021). Getting to know you: Emerging neural representations during face familiarization. Journal of Neuroscience.

[CR2] Andrews S, Jenkins R, Cursiter H, Burton AM (2015). Telling faces together: Learning new faces through exposure to multiple instances. Quarterly Journal of Experimental Psychology.

[CR3] Bindemann M, Attard J, Leach A, Johnston RA (2013). The effect of image pixelation on unfamiliar-face matching. Applied Cognitive Psychology.

[CR4] Bindemann M, Burton AM, Jenkins R (2005). Capacity limits for face processing. Cognition.

[CR5] Bonner L, Burton AM, Jenkins R, McNeill A, Vicki B (2003). Meet The Simpsons: Top-down effects in face learning. Perception.

[CR6] Bruce V, Young A (1986). Understanding face recognition. British Journal of Psychology.

[CR100] Bruce, V., Henderson, Z., Greenwood, K., Hancock, P. J. B., Burton, A. M., & Miller, P. (1999). Verification of face identities from images captured on video.* Journal of Experimental Psychology: Applied, 5*(4), 339-360. 10.1037/1076-898X.5.4.339.

[CR7] Burton AM, Bruce V, Johnston RA (1990). Understanding face recognition with an interactive activation model. British Journal of Psychology.

[CR101] Burton, A. M., White, D., & McNeill, A. (2010). The Glasgow face matching test.* Behavioural Research Methods, 42*(1), 286–291. 10.3758/BRM.42.1.286. 10.3758/BRM.42.1.286 20160307

[CR8] Davis JP, Valentine T (2009). CCTV on trial: Matching video images with the defendant in the dock. Applied Cognitive Psychology.

[CR9] Dror IE, Charlton D, Péron AE (2006). Contextual information renders experts vulnerable to making erroneous identifications. Forensic Science International.

[CR10] Dror IE, Péron AE, Hind S, Charlton D (2005). When emotions get the better of us: The effect of contextual top-down processing on matching fingerprints. Applied Cognitive Psychology.

[CR11] Dror IE, Thompson WC, Meissner CA, Kornfield I, Krane D, Saks M, Risinger M (2015). Letter to the editor− Context management toolbox: A linear sequential unmasking (LSU) approach for minimizing cognitive bias in forensic decision making. Journal of Forensic Sciences.

[CR12] Dunn JD, Kemp RI, White D (2021). Top-down influences on working memory representations of faces: Evidence from dual-target visual search. Quarterly Journal of Experimental Psychology.

[CR13] Edmond G, Kemp R, Porter G, Hamer D, Burton M, Biber K, Roque MS (2010). Atkins v The Emperor: The ‘cautious’ use of unreliable ‘expert’ opinion. The International Journal of Evidence and Proof.

[CR14] Faul F, Erdfelder E, Lang A, Buchner A (2007). G*Power 3: A flexible statistical power analysis program for the social, behavioral, and biomedical sciences. Behavior Research Methods.

[CR15] Feng X, Burton AM (2019). Identity documents bias face matching. Perception.

[CR16] Feng X, Burton AM (2021). Understanding the document bias in face matching. Quarterly Journal of Experimental Psychology.

[CR17] Fysh MC, Bindemann M (2018). Human-computer interaction in face matching. Cognitive Science.

[CR18] Heekeren HR, Marrett S, Ungerleider LG (2008). The neural systems that mediate human perceptual decision making. Nature Reviews Neuroscience.

[CR19] Howard JJ, Rabbitt LR, Sirotin YB (2020). Human-algorithm teaming in face recognition: How algorithm outcomes cognitively bias human decision-making. PLoS ONE.

[CR20] Jenkins R, Lavie N, Driver J (2003). Ignoring famous faces: Category-specific dilution of distractor interference. Perception and Psychophysics.

[CR21] Johnston A, Hill H, Carman N (1992). Recognising faces: Effects of lighting direction, inversion, and brightness reversal. Perception.

[CR102] Karimi-Rouzbahani, H., Ramezani, F., Woolgar, A., Rich, A., & Ghodrati, M. (2021). Perceptual difficulty modulates the direction of information flow in familiar face recognition.* Neuroimage, 233*, 117896. 10.1016/j.neuroimage.2021.117896. 10.1016/j.neuroimage.2021.117896 PMC7614447 33667671

[CR22] Kaufmann JM, Schweinberger SR, Burton AM (2008). N250 ERP correlates of the acquisition of face representations across different images. Journal of Cognitive Neuroscience.

[CR23] Klink PC, van Wezel RJ, van Ee R (2012). United we sense, divided we fail: Context-driven perception of ambiguous visual stimuli. Philosophical Transactions of the Royal Society B.

[CR24] Kovacs G (2020). Getting to know someone: Familiarity, person recognition, and identification in the human brain. Journal of Cognitive Neuroscience.

[CR25] Li S, Yang F (2012). Task-dependent uncertainty modulation of perceptual decisions in the human brain. European Journal of Neuroscience.

[CR26] Maksimenko VA, Kuc A, Frolov NS, Khramova MV, Pisarchik AN, Hramov AE (2020). Dissociating cognitive processes during ambiguous information processing in perceptual decision-making. Frontiers in Behavioural Neuroscience.

[CR27] McCaffery JM, Burton AM (2016). Passport checks: Interactions between matching faces and biographical details. Applied Cognitive Psychology.

[CR28] Menon N, White D, Kemp RI (2015). Identity-level representations affect unfamiliar face matching performance in sequential but not simultaneous tasks. Quarterly Journal of Experimental Psychology.

[CR29] Noyes E, Jenkins R (2017). Camera-to-subject distance affects face configuration and perceived identity. Cognition.

[CR30] Oliva A, Torralba A (2007). The role of context in object recognition. Trends in Cognitive Sciences.

[CR31] Peirce JW, MacAskill MR (2018). Building experiments in PsychoPy.

[CR32] Porter G (2009). CCTV images as evidence. Australian Journal of Forensic Sciences.

[CR33] Qi S, Footer O, Camerer CF, Mobbs D (2018). A collaborator's reputation can bias decisions and anxiety under uncertainty. Journal of Neuroscience.

[CR34] Ramon M, Miellet S, Dzieciol AM, Konrad BN, Dresler M, Caldara R (2016). Super-memorizers are not super-recognizers. PLoS ONE.

[CR35] Robertson DJ, Burton AM (2021). Checking ID-cards for the sale of restricted goods: Age decisions bias face decisions. Applied Cognitive Psychology.

[CR36] Schwartz L, Yovel G (2016). The roles of perceptual and conceptual information in face recognition. Journal of Experimental Psychology: General.

[CR37] Schwartz L, Yovel G (2019). Learning faces as concepts rather than percepts improves face recognition. Journal of Experimental Psychology: Learning, Memory, and Cognition.

[CR38] Shoham A, Kliger L, Yovel G (2021). Learning faces as concepts improves face recognition by engaging the social brain network. Social Cognitive and Affective Neuroscience.

[CR39] Smalarz L, Madon S, Yang Y, Guyll M, Buck S (2016). The perfect match: Do criminal stereotypes bias forensic evidence analysis?. Law and Human Behavior.

[CR40] Stanislaw H, Todorov N (1999). Calculation of signal detection theory measures. Behavior Research Methods, Instruments, and Computers.

[CR41] Sternberg S (1969). The discovery of processing stages: extensions of Donders’ method. Acta Psychologica.

[CR42] Sternberg S (2011). Modular processes in mind and brain. Cognitive Neuropsychology.

[CR43] Todorov A, Gobbini MI, Evans KK, Haxby JV (2007). Spontaneous retrieval of affective person knowledge in face perception. Neuropsychologia.

[CR44] Trapp S, Schweinberger SR, Hayward WG, Kovacs G (2018). Integrating predictive frameworks and cognitive models of face perception. Psychonomic Bulletin and Review.

[CR45] Walker H, Tough A (2015). Facial comparison from CCTV footage: The competence and confidence of the jury. Science and Justice.

[CR46] White D, Kemp RI, Jenkins R, Matheson M, Burton AM (2014). Passport officers’ errors in face matching. PLoS ONE.

[CR47] White D, Phillips PJ, Hahn CA, Hill M, O'Toole AJ (2015). Perceptual expertise in forensic facial image comparison. Proceedings of the Royal Society B: Biological Sciences.

[CR48] White D, Towler A, Kemp RI, Bindemann M (2020). Understanding professional expertise in unfamiliar face matching. Forensic face matching: Research and practice.

[CR49] Wurm MF, Schubotz RI (2017). What's she doing in the kitchen? Context helps when actions are hard to recognize. Psychonomic Bulletin and Review.

[CR50] Young AW, Ellis AW, Flude BM, McWeeny KH, Hay DC (1986). Face–name interference. Journal of Experimental Psychology: Human Perception and Performance.

