# Supplementary material for: Verifying unfamiliar identities: Effects of processing name and face information in the same identity-matching task
Source: Cogn Res Princ Implic. 2022 Oct 12;7:92. doi: 10.1186/s41235-022-00441-2 (PMC9556678; doi:10.1186/s41235-022-00441-2)
Supplement: Supplementary file 1 — Additional file 1. Supplementary Materials. [file 41235_2022_441_MOESM1_ESM.pdf]

Verifying unfamiliar identities: Effects of processing name and face information in the same identity-matching task

Supplementary Materials

Anita Trinh, James D. Dunn, David White

Corresponding Author: David White (david.white@unsw.edu.au)

Sensitivity Analysis

For Experiments 1-3 we analysed face matching performance using signal detection measures of sensitivity and criterion (Stanislaw & Todorov, 1999). This measure was included to ensure that the image quality manipulation was successful in increasing the difficulty of the matching task. While criterion was the primary measure of interest, here we present the results of our analysis of sensitivity for each experiment.

In summary, across all experiments we found a consistent and expected main effect of image quality on sensitivity, in that a lower image quality led to significantly reduced sensitivity scores. The summary graph containing sensitivity scores for all experiments which manipulated image quality can be found in Figure S1 below. Details of sensitivity scores across the four experiments (including the Experiment 2 pilot study), in numerical experiment order, can be found in Figure S2, Figure S8, Figure S3, and Figure S4 respectively.

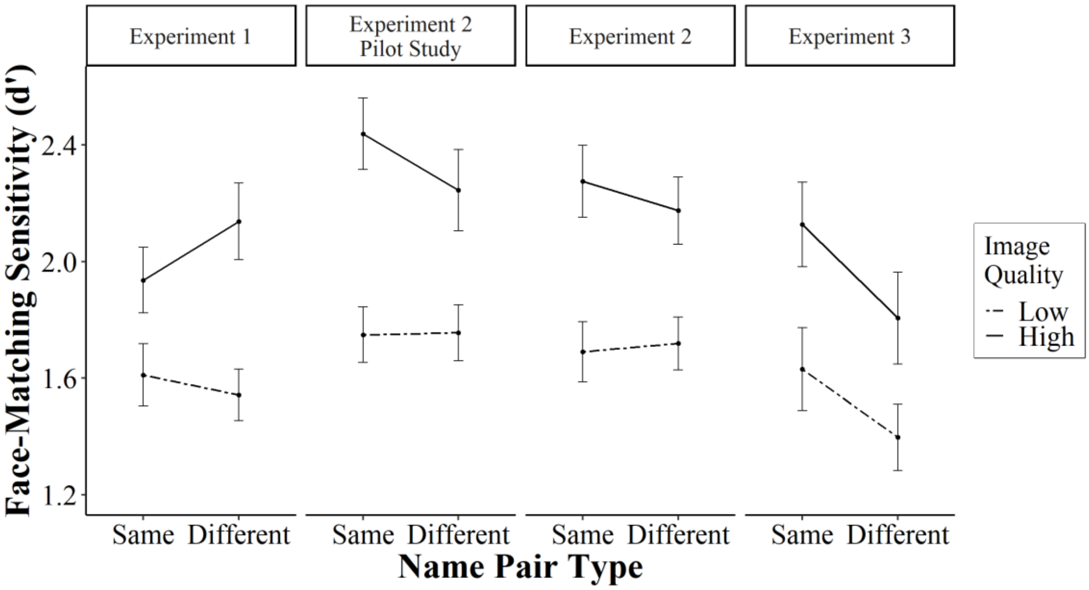

Figure S1. Face-matching sensitivity (d') scores as a function of image quality and name pair type for Experiment 1 (name first condition only), the Experiment 2 pilot study, Experiment 2 (name first condition only), and Experiment 3. All error

bars represent standard error. It was consistently found that having a facial comparison image with low image quality significantly decreased face-matching sensitivity. This consistent main effect of image quality on face-matching sensitivity demonstrates that our manipulation of blurring one of two images was effective in increasing the visual difficulty of the face matching task.

Experiment 1

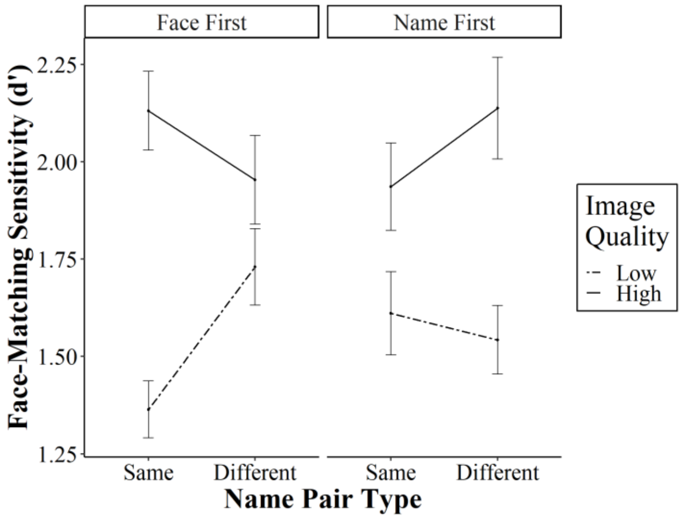

Figure S2. Experiment 1 sensitivity (d') scores across factors of image quality and context type for face first and name first conditions. All error bars represent standard error.

For both between-subjects conditions, participants were better at discriminating between match and non-match trials when image quality was high (face first: $F_{1, 45}$= 36.17, $p < 0.005$, $\eta p^2 = 0.45$; name first: $F_{1, 45} = 31.23$, $p < 0.005$, $\eta p^2 = 0.09$). Name pair type did not affect sensitivity (face first: $F_{1, 45}$= 1.24, $p = 0.27$, $\eta p^2 = 0.03$; name first: $F_{1, 45}$= 0.70, $p = 0.40$, $\eta p^2 = 0.02$).

There was a significant interaction between face trial type and image quality in the face first condition ($F_{1, 45} = 13.39$, $p < 0.005$, $\eta p^2 = 0.23$); simple-effects analysis shows that the interaction is driven by a significantly lower sensitivity for "same" compared to "different" name pair types in the low image quality condition ($F_{1, 45} = 13.66$, $p < 0.005$, $\eta p^2 = 0.23$). However, there was no significant interaction between image quality and face trial type in the name first condition ($F_{1, 45} = 2.31$, $p = 0.14$, $\eta p^2 = 0.04$). Given that a sensitivity interaction is only observable in the face first condition for Experiment 1 and is not in subsequent experiments (see Figure S3 and Figure S4), we do not believe that the sensitivity interaction is of theoretical significance.

Experiment 2

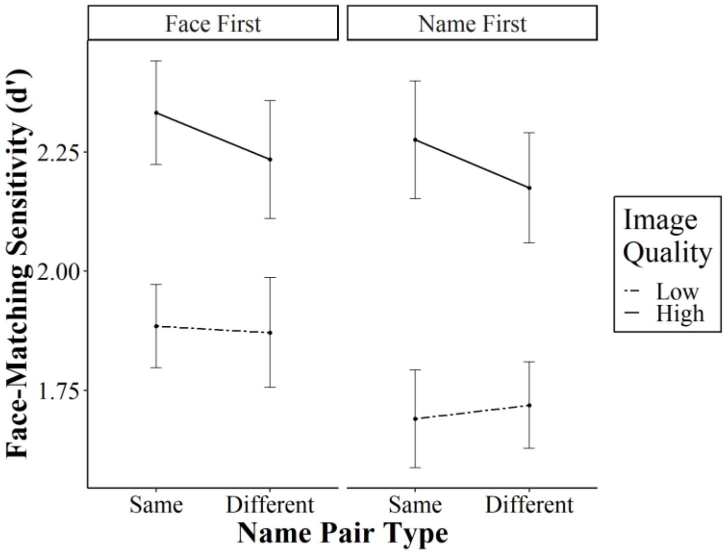

Figure S3. Experiment 2 face-matching sensitivity (d') scores as a function of image quality and name pair type. All error bars represent standard error.

As observed in previous experiments, participants were poorer at distinguishing between match and non-match face trials when image quality was low (face first: $F_{1, 34} = 27.87$, $p < 0.005$, $\eta p^2 = 0.45$; name first: $F_{1, 30} = 21.65$, $p < 0.005$, $\eta p^2 = 0.42$). There were no effects of name pair type on sensitivity (face first: $F_{1, 34} = 0.22$, $p = 0.64$, $\eta p^2 = 0.006$; name first: $F_{1, 30} = 0.08$, $p = 0.78$, $\eta p^2 = 0.002$) and no interaction between image quality and face trial type on sensitivity (face first: $F_{1, 34} = 0.18$, $p = 0.68$, $\eta p^2 = 0.005$; name first: $F_{1, 30} = 0.43$, $p = 0.51$, 0.01).

Experiment 3

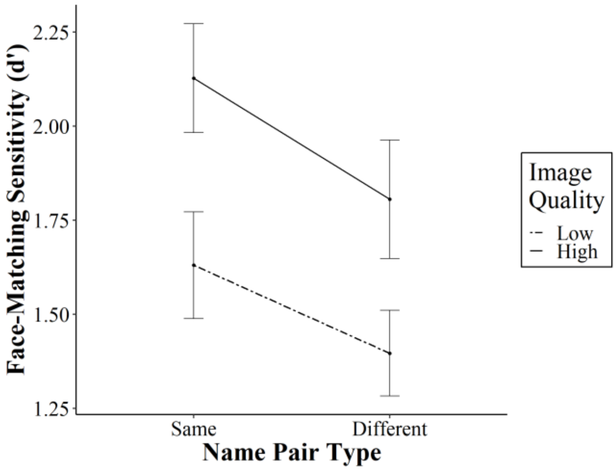

Figure S4. Experiment 3 face-matching sensitivity (d') scores as a function of image quality and name pair type. All error bars represent standard error.

As expected, there was a significant effect of image quality on face-matching sensitivity ($F_{1,42} = 29.97$, $p < 0.001$, $\eta p^2 = 0.42$) whereby participants were better able to distinguish between face pairs of higher quality. However, there was also an unexpected effect of name pair type on sensitivity ($F_{1,42} = 4.41$, $p = 0.04$, $\eta p^2 = 0.10$), in that participants had reduced sensitivity for face pairs that followed different name pairs. There was no interaction between image quality and name pair type ($F_{1,42} = 0.25$, $p = 0.62$, $\eta p^2 = 0.006$).

Post-hoc Analysis: Previous Name Pair Type

Experiment 1

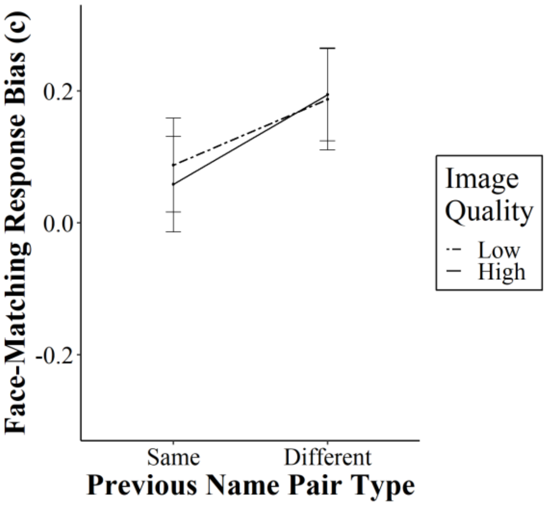

Figure S5. Experiment 1 response bias (c) across factors of image quality and previous name pair type for the face-first condition only. All error bars represent standard error.

The purpose of a post-hoc analysis for Experiment 1 was to assess whether the face-matching response bias, observed in the name-first condition, was generated by name pairs that pertained to the facial identities, or whether the bias resulted simply from name pairs from any preceding decision. A post-hoc analysis was conducted on response bias scores from the face-first condition where the face matching decision had been preceded by a name matching decision *in the previous trial*. We conducted a 2 × 2 ANOVA on the resulting data across levels of previous name pair type and image quality.

As observed in Figure S5, there is a significant main effect of previous name pair type on response biases ($F_{1,\,45} = 4.37$, $p = 0.04$, $\eta p^2 = 0.09$) whereby participants completing the face-first condition were more biased towards making a “match” face decision when the previous identity trial presented matching names.

Experiment 2

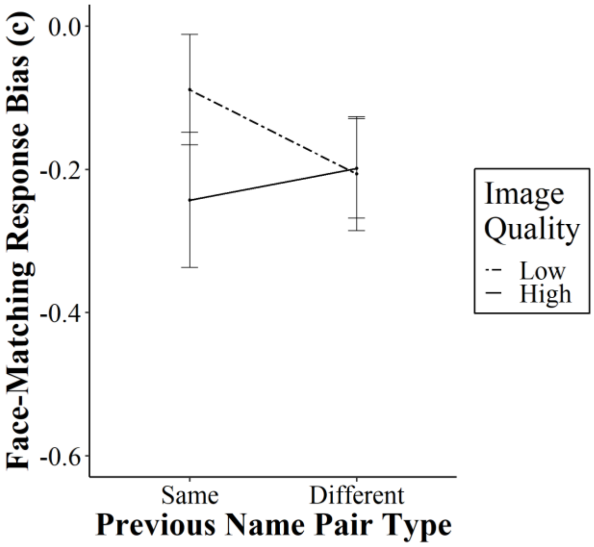

Figure S6. Experiment 2 face-matching response bias (c) scores across factors of previous name pair type and image quality. All error bars represent standard error.

The same post-hoc analysis applied to Experiment 1 was conducted for the face-first condition data in Experiment 2, to assess whether the ID frame manipulation confined the face-matching response bias to a per-identity (or per-trial) level. These results of the ANOVA are shown in Figure S6. In contrast to Experiment 1, we found no effect of previous name pair type on response bias for face-matching decisions for Experiment 2 ($F_{1, 34} = 0.47$, $p = 0.50$, $\eta p^2 = 0.01$).

Response Biases

For illustrative purposes, a summary graph of response biases across levels of name pair type and image quality, for Experiments 1 to 3 including pilot studies, can be found in Figure S7. Notably, the difference in magnitude of the response bias effect between Experiment 2 ($\eta p^2 = 0.15$) and Experiment 3 ($\eta p^2 = 0.27$) is clearly visible. This effect size difference is likely to be a result of the change in instructions across the two experiments – whereas participants were told to ignore name information in their face-matching decisions in Experiment 2, participants were falsely informed of a predictive relationship between name pair types and face trial types in Experiment 3.

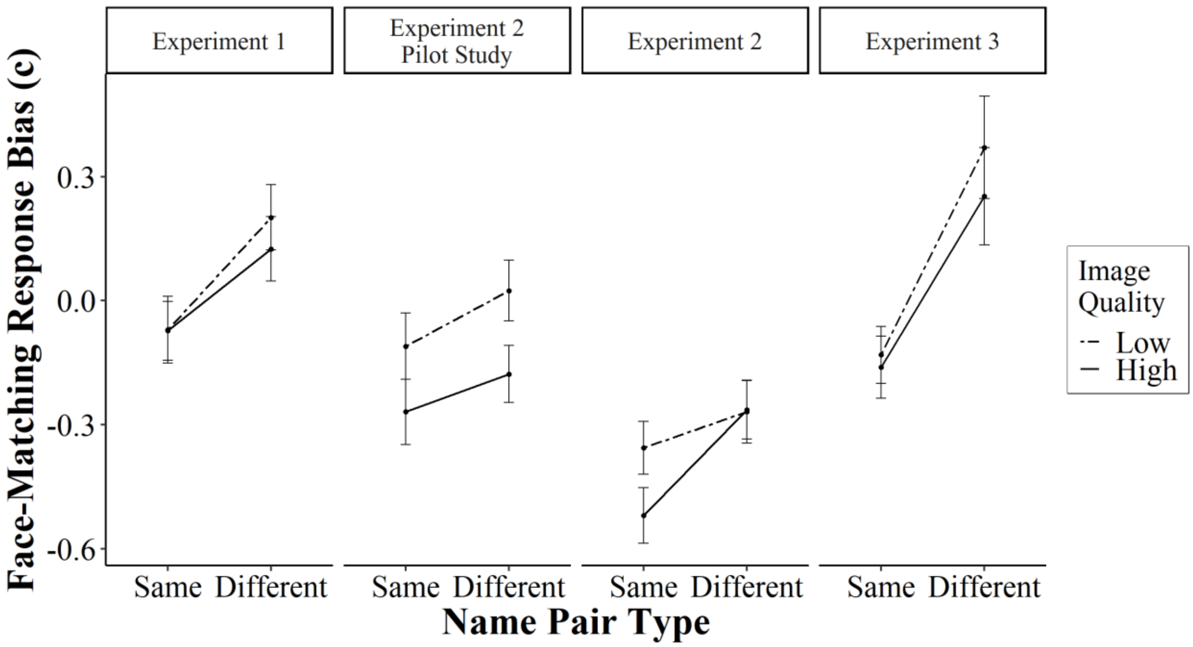

Figure S7. Face-matching response bias (c) scores as a function of image quality and name pair type for Experiment 1 (name first condition only), the Experiment 2 pilot study, Experiment 2 (name first condition only), and Experiment 3. All error bars represent standard error. Across all four experiments, there was a significantly greater bias towards "match" decisions in face-matching trials preceded by matching name pairs.

Pilot Experiments

Experiment 2 (Pilot Study)

We conducted a pilot study that was very similar to Experiment 2. This pilot only used a name-first design, contained slight differences in the task instructions, and participants were given trial-by-trial feedback on their accuracy (“correct” or “incorrect”). The results of this study were very similar to Experiment 2 and so are presented in Supplementary Material along with a detailed description of the study method.

Participants

Sixty undergraduate students from UNSW Sydney participated in the Experiment 2 pilot study for course credit (M = 31, F = 29). Two participants were excluded due to non-completion of the experiment, leaving a total of 58 participants in the final analysis (30 male, M_{age} = 20.0 years, SD_{age} = 2.7 years). Participants scored an average of 79.3% for the face matching decisions (SD = 7.7%). All participants scored above 90% for the name decisions with an average performance of 98.5% (SD = 1.6%).

Design and Procedure

The facial stimuli used in the Experiment 2 pilot study are the same as that used in Experiment 1. The experimental design for the pilot study is the same as the design for Experiment 2 (reported in the main paper), but for a few main differences: a “face-first” task order condition was not included, and participants were not given any instructions regarding the predictability of name information on face matches.

Results

As in Experiment 1, we conducted a 2 × 2 ANOVA for sensitivity and criterion scores across factors of image quality and name pair type.

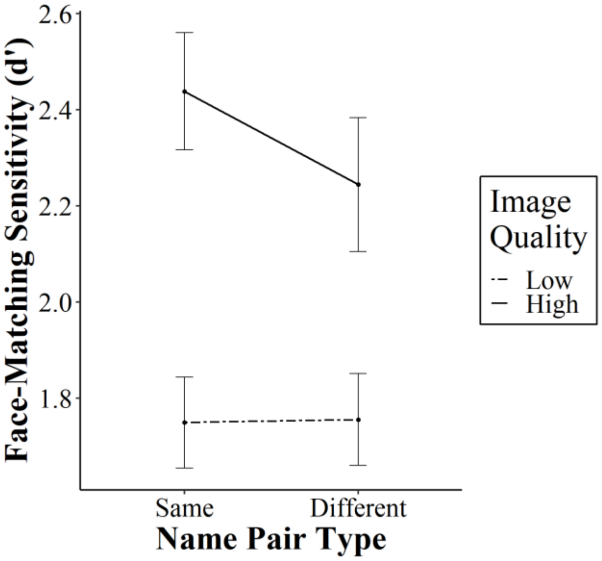

Figure S8. Experiment 2 pilot study sensitivity (d’) scores across factors of image quality and context type. All error bars represent standard error.

Face-matching sensitivity was significantly lower when one of two facial images were blurred ($F_{1,\,57}$= 48.49, $p < 0.005$, $\eta p^2 = 0.104$). There was no significant effect of name pair type on sensitivity ($F_{1,\,57}$= 1.17, $p = 0.28$, $\eta p^2 = 0.02$), nor was there an interaction of sensitivity between image quality and name pair type ($F_{1,\,57}$= 1.65, $p = 0.20$, $\eta p^2 = 0.03$).

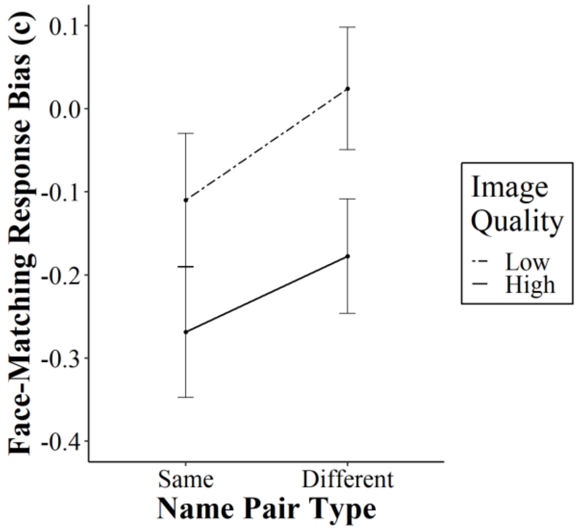

Figure S9. Experiment 2 pilot study response bias (c) scores across factors of image quality and name pair type. All error bars represent standard error.

We found a significant effect of name pair type ($F_{1,\ 57}$= 7.46, p = 0.008, ηp^2 = 0.12) on face matching response biases, with matching name pairs making participants more likely to make match responses to the subsequent face matching decision. However, unlike Experiment 1, we also observed a significant main effect of image quality on response bias, ($F_{1,\ 57}$= 21.31, p < 0.005, ηp^2 = 0.27), with face matching skewed towards "match" responses when the image quality was high. This result is difficult to explain post-hoc, given that the new visual changes implemented in the pilot study applied to *all* face matching decisions, regardless of its within-subject condition. Image quality did not interact with name pair type ($F_{1,\ 57}$ = 0.35, p = 0.56, ηp^2 = 0.006).

Experiment 4 (Pilot Study)

A pilot study of Experiment 4 was also conducted. In the pilot study, the order in which participants completed information type blocks was fully randomised – otherwise, the study design is identical to that of Experiment 4. In Experiment 4, participants were randomly allocated a set order within which information type matches would be completed (name first, date first, or object first).

Participants

Forty-one participants from UNSW Sydney participated in the study (13 male, M_{age} = 19.5 years). Participants were excluded from the pilot study data analysis if their context-matching accuracy was below 95% and if their face matching accuracy was below 50%. Only thirty-seven participants were included in the data analysis after removing four participants (12 male, M_{age} = 19.6 years, SD_{age} = 2.5 years). Undergraduate participants were recruited online and completed the experiment on Pavlovia (Peirce & MacAskill, 2018) using their personal computers.

Design and Procedure

The facial stimuli, procedure, and written information presented in this study is identical to that of Experiment 4 (reported in the main paper). However, in the pilot study, the order in which information type blocks were presented was completely randomised for each participant.

Results

Participants performed at 77.6% accuracy for face matching decisions (SD = 9.4%). Following participant exclusions, the average accuracy for name, date, and object-matching decisions were 98.1% (SD = 2.1%), 99.5% (SD = 0.8%) and 99.4% (SD = 1.7%) respectively.

A 3 × 2 ANOVA was conducted for criterion scores across factors of Pair Type (same, different) and Information Type (name, date, object). Given that the image quality manipulation is no longer included in the experimental design, we did not compute sensitivity scores. The results of the response bias ANOVA are shown in Figure S10.

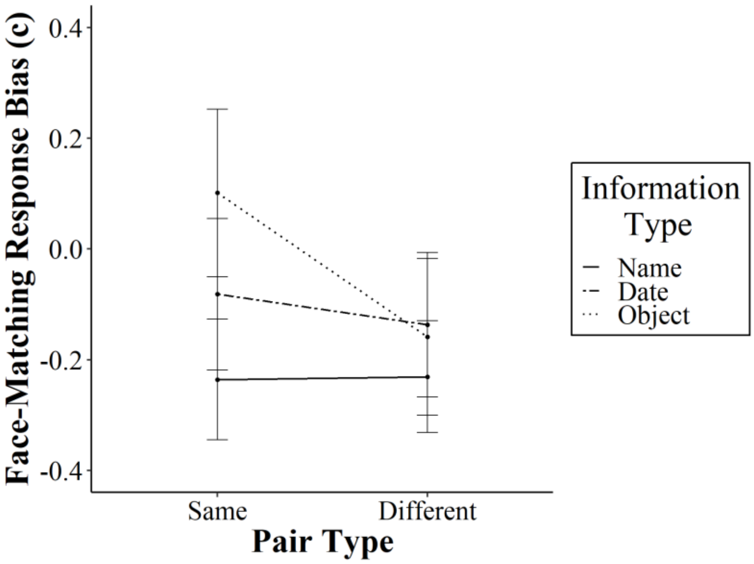

Figure S10. Face-matching response bias (c) scores in the Experiment 4 pilot study as a function of pair type and information type. All error bars represent standard error.

There was a significant main effect of pair type on face matching response biases ($F_{1, 36}$ = 4.40, *p* = 0.04, ηp^2 = 0.11), albeit in an unexpected direction; participants were significantly more likely to respond "non-match" in face matching trials preceded by the *same* pair type. There was no main effect of information type on face matching response biases ($F_{1, 72}$ = 1.25, *p* = 0.29, ηp^2 = 0.03); however, there was a significant interaction in criterion scores between information type and pair type ($F_{1, 72}$ = 3.31, *p* = 0.04, ηp^2 = 0.08). This interaction was driven by the object information type, whereby there was a greater "non-match" response bias in face trials when they were preceded by the *same* pair type ($F_{1, 36}$ = 8.86, *p* = 0.005, ηp^2 = 0.20). Unexpectedly, there was no significant difference in criterion scores between pair type levels within the name ($F_{1, 36}$ = 0.004, *p* = 0.95, ηp^2 < 0.001) or date ($F_{1, 36}$ = 0.59, *p* = 0.45, ηp^2 = 0.02) information types. In other words, the main response bias effect of name pair type on face-matching response biases, seen across Experiments 1 to 3, was not observed in the Experiment 4 pilot study.

Given that we did not observe the predicted same-face response bias for matching pairs in the name condition, we theorised that the order in which information types were completed had some influence over face matching response bias patterns. To test this theory with our Experiment 4 pilot data, we generated an "information type order" between-subjects variable for each participant based on the information type assigned to their first trial. We then performed a three-way mixed models ANOVA with criterion scores across factors of pair

type, information type, and information type order. The results of this ANOVA are detailed in Figure S11. We note that there was an uneven number of participants in each between-subjects condition (name first: n = 12, date first: n = 14, object first: n = 11). This was to be expected, given that each participant was assigned a fully randomised order in which to complete information type matching.

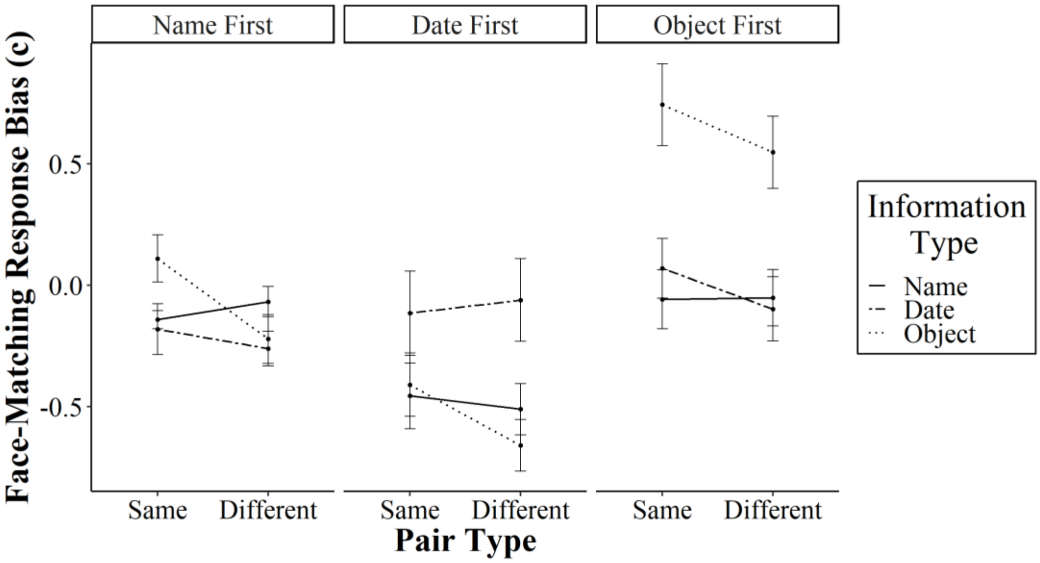

Figure S11. A post-hoc analysis of data from the pilot study for Experiment 4 across factors of pair type, information type and information type order (a factor which was created post-hoc based on the information type presented first for each participant). All error bars represent standard error.

Our theory that information type order had an influence over response bias patterns could not be confirmed by our ANOVA. We found no significant main effect of information type order on face-matching response biases ($F_{2, 34}$ = 3.04, *p* = 0.06, ηp^2 = 0.15), and no significant interaction between information type order and pair type ($F_{2, 34}$ = 0.04, *p* = 0.95, ηp^2 = 0.003). However, a visual observation of response bias scores in the "object first" condition (as seen in Figure S11) appears consistent with our theory that information type order may be influencing how different information types were matched. The uneven participant allocation to condition orders, as well as the small sample size, may have affected our ability to detect any statistical between-subjects differences across information type order in this experiment.

In Experiment 4, we systematically varied the order in which participants completed the information type matching. Participants either completed the name, date, or object information conditions first, and the order in which subsequent blocks were completed were pre-defined based on the order allocation (e.g., all participants who completed the “name”

information type matching first were then given the “date” and “object” information types respectively). We also recruited a larger number of participants compared to the Experiment 4 pilot study for greater statistical power in analysing the effect of block order conditions on face-matching response biases. To check whether the order of information type completion affected response biases, we repeated the 3 x 2 ANOVA (as conducted for the Experiment 4 pilot study) across factors of information type order, information type, and pair type. The results of this analysis are shown in Figure S12.

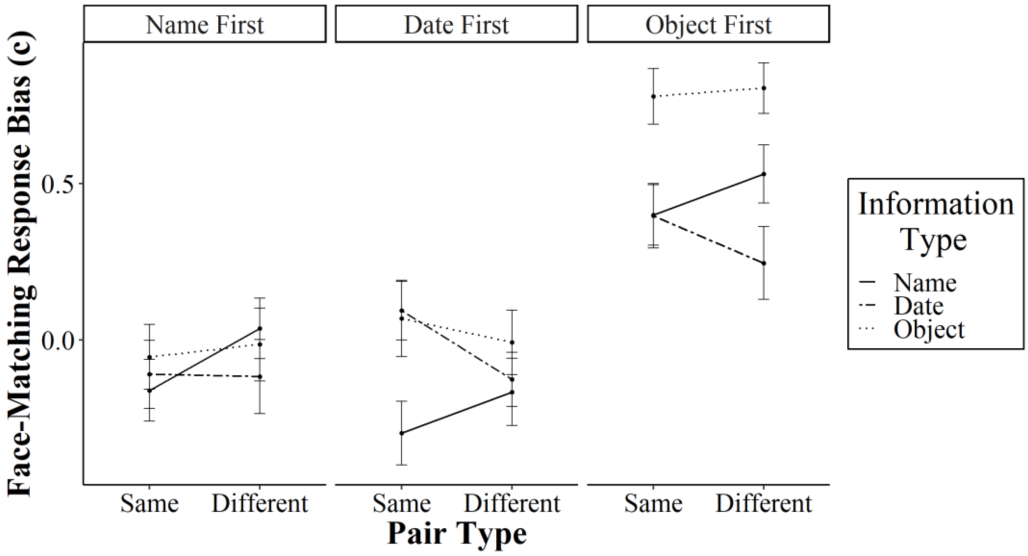

Figure S12. Experiment 4 face-matching response bias (c) scores as a function of pair type and information type, split across levels of information type order. All error bars represent standard error.

There was a significant main effect of information type order on face-matching response biases ($F_{2, 70} = 4.57$, $p = 0.01$, $\eta p^2 = 0.12$), which aligned with our prediction following the Experiment 4 pilot study. Similarities in response bias trends can be observed within the “object first” condition between Figure S11 and Figure S12. The main effect was driven by a significantly greater non-match response bias for faces in the object first condition compared to the name first ($F_{1, 46} = 7.19$, $p = 0.01$, $\eta p^2 = 0.14$) and date first ($F_{1, 46} = 7.02$, $p = 0.01$, $\eta p^2 = 0.13$) conditions. There was no statistical difference in response bias scores between the name first and date first conditions ($F_{1, 48} < 0.001$, $p = 0.99$, $\eta p^2 < 0.001$). These results confirmed our theory that the order in which information types were completed affected the response bias scores within each information type condition. We suspect that a difference in instructions for the object first condition may have led to a base shift in response criterion for participants completing the experimental tasks (details of instructional differences have been detailed in the main paper).

Stimuli

Experiment 4

A list of object words used for the Experiment 4 “object” information type is included below (in randomised order):

1. OCEAN
2. COMPUTER
3. SPOON
4. NOODLES
5. ROAD
6. BILL
7. ARTWORK
8. WRIST
9. CARPET
10. LIGHTER
11. BOAT
12. CABIN
13. XYLOPHONE
14. FREEZER
15. HEELS
16. STOVE
17. WINGS
18. JUMPER
19. CANE
20. JACKET
21. PIPE
22. BALLOON
23. CONDITIONER
24. CANDY
25. GRAPES
26. TOY
27. FOOT
28. AIRCRAFT
29. SUNGLASSES
30. VILLAGE
31. RING
32. BLENDER
33. SHIP
34. WATER
35. VILLA
36. SANDALS
37. SUITCASE
38. MOONLIGHT
39. CANOE
40. FINGER
41. BREAD
42. SANDWICH
43. VEST
44. SLIPPERS
45. SOFA
46. SOAP
47. COOKIE
48. TRAIN
49. BEANS
50. SNOWMAN
51. VOICE
52. SPAGHETTI
53. ESTATE
54. TOES
55. TISSUE
56. WATCH
57. COUNTRY
58. SHAMPOO
59. PURSE
60. KNIFE
61. PAD
62. OIL
63. BOARD
64. TRUCK
65. SHOWER
66. TOOTHBRUSH
67. SUN
68. SAND
69. BANK
70. BUTTER
71. ONION
72. TAXI
73. GARLIC
74. TABLET
75. JEWELRY
76. BULB
77. CLOCK
78. COUCH
79. PALM
80. REFRIGERATOR
81. CAP
82. JERSEY
83. TEMPLE
84. WOOL
85. CELLO
86. NEWSPAPER
87. BRASS
88. TEA
89. PANCAKE
90. TELEVISION
91. TUNNEL
92. FILM
93. ARROW
94. PANTS
95. PIANO
96. RADIO
97. GIFT
98. SALT
99. SCONES
100. CARDIGAN
101. ANKLE
102. SKYSCRAPER
103. RIBS
104. SMOKE
105. PERFUME
106. PEN
107. NECKLACE
108. MEDICINE
109. LUGGAGE
110. EARRINGS
111. BOOTS
112. APARTMENT
113. TROUSERS
114. SHIRT
115. STAR
116. MUSEUM
117. COOKWARE
118. OVEN
119. BAG
120. SKY
121. VEHICLE
122. PYRAMID
123. TIE
124. PASTA
125. ORANGE
126. TREE
127. WATERMELON
128. GUITAR
129. TOMATO
130. CHAIR
131. GLOVES
132. BLAZER
133. RULER
134. FAN
135. FARM
136. SUPERMARKET
137. ANTLERS
138. FOREST

139. MALL
140. THEATER
141. SCISSORS
142. REMOTE
143. STREET
144. VASE
145. LAMP
146. BOOK
147. BOW
148. SUIT
149. GALAXY
150. SWEATER
151. STOMACH
152. STRAWBERRY
153. UMBRELLA
154. CAVE
155. LEG
156. KEYBOARD
157. SKIRT
158. UNDERWEAR
159. MICROSCOPE
160. DESK
161. PUMPKIN
162. EGG
163. CABINET
164. BOOKCASE
165. HEART
166. PLANE
167. FORK
168. BOXERS
169. SOCKS
170. CRIB
171. FROCK
172. BLOUSE
173. COFFEE
174. RAINBOW
175. MOBILE
176. LUNG
177. BUCKLES
178. WHISKER
179. WALLET
180. PLANT
181. HOTEL
182. TAIL
183. MUSIC
184. LEMON
185. POPCORN
186. NOTEBOOK
187. LIBRARY
188. PAPER
189. PHONE
190. SCALE
191. APPLE
192. HERBS
193. ROOF
194. DRESS
195. HOUSE
196. LAPTOP
197. BALL
198. TELEPHONE
199. GRAINS
200. BUCKET
201. SHORTS
202. WAIST
203. TOOTHPASTE
204. POTATO
205. STADIUM
206. GOWN
207. TONGUE
208. TOWN
209. HAMMER
210. RESTAURANT
211. DESKTOP
212. STAIRS
213. MIRROR
214. BAT
215. CHEESE
216. DRESSER
217. SHOES
218. NOISE
219. CAR
220. PARK
221. FACTORY
222. CHEST
223. POUCH
224. METAL
225. PAGODA
226. AMBULANCE
227. HAND
228. FLOWER
229. MARKET
230. BED
231. SAXOPHONE
232. HAMBURGER
233. SHRIMP
234. CHOCOLATE
235. FRUIT
236. VIOLIN
237. BRIDGE
238. PENCIL
239. CREST
240. BELT
241. ALLIGATOR
242. BACK
243. CASTLE
244. ARMCHAIR
245. MANGO
246. SINK
247. SOUP
248. DRUM
249. FLAG
250. COSTUME
251. GARAGE
252. TABLE

References

Feng, X., & Burton, A. M. (2019). Identity Documents Bias Face Matching. *Perception, 48*(12), 1163-1174. https://doi.org/10.1177/0301006619877821

McCaffery, J. M., & Burton, A. M. (2016). Passport checks: Interactions between matching faces and biographical details. *Applied Cognitive Psychology, 30*, 925-933. https://doi.org/10.1002/acp.3281

Peirce, J. W., & MacAskill, M. R. (2018). *Building experiments in PsychoPy*. Sage.
